# Vitamin A deficiency triggers colonic methylation potentially impairing colonic neuron via downregulation SGK1/FOXO pathway

**DOI:** 10.1002/pdi3.86

**Published:** 2024-06-14

**Authors:** Bei Tong, Junyan Yan, Zhujun Sun, Ruifang Luo, Fang Lin, Riqiang Hu, Ting Yang, Yuting Wang, Jie Chen

**Affiliations:** ^1^ Children Nutrition Research Center Children's Hospital of Chongqing Medical University Chongqing Key Laboratory of Child Neurodevelopmental and Cognitive Disorders Ministry of Education Key Laboratory of Child Development and Disorders National Clinical Research Center for Child Health and Disorders Chongqing China; ^2^ Department of Gastroenterology Children's Hospital of Chongqing Medical University Chongqing China

**Keywords:** enteric neurons, methylation, SGK1/FOXO pathway, vitamin A supplementation, vitamin A deficiency

## Abstract

DNA methylation is widely involved in the modification of intestinal function, but the methylation mechanism in the enteric nervous system has not been studied in vitamin A deficiency (VAD). Herein, we firstly found that in the VAD group, gastrointestinal transit time was delayed compared with the vitamin A normal (VAN) group. RNA sequencing between VAD and VAN rats identified enriched pathways associated with enteric nerves and methylation transferase complexes. Then expression levels of DNA methyltransferases (DNMT1, DNMT3a and DNMT3b) were validated to significant increase in the VAD group. Representative reduced bisulfate sequencing showed that the VAD rats had high levels of DNA methylation in promoters and exons compared with the VAN rats. A combined methylomic and transcriptomic analysis identified that methylation levels of *Sgk1*, a key gene associated with enteric neural development, were elevated in the VAD group, and the activity of the SGK1/FOXO signaling axis was reduced. Furthermore, the colonic neuronal morphology and synaptic architecture were impaired in the VAD offspring. Interestingly, the above alterations in the VAD group were alleviated by vitamin A (VA) supplementation in the early postnatal period. These data suggest that VAD triggers colonic hypermethylation, which probably downregulates the SGK1/FOXO signaling pathway to cause colonic transfer dysfunction.

## Graphical abstract

Colonic hypermethylation is shown in the vitamin A deficiency (VAD) pups. VAD damages synaptic structures and impairs colonic neuron morphology. Downregulated SGK1‐FOXO pathway may contribute to enteric neuron dysfunction.

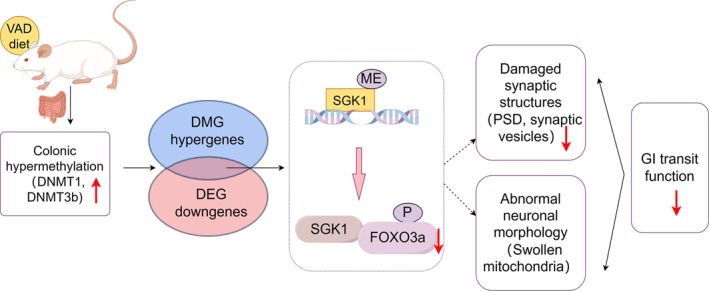

## 1 INTRODUCTION

Vitamin A deficiency (VAD) is one of the four major nutritional deficiencies recognized by the World Health Organization and is most common in infants and young children.^1^ Using vitamin A (VA) derivatives, a study showed that VAD can affect intestinal digestion and absorption by disrupting intestinal integrity.^2^ VAD also leads to dysbiosis of intestinal microbial ecosystems, exacerbation of colitis, and increased susceptibility to gastrointestinal (GI) infections.^3^ This is in agreement with the findings of our group that VAD during pregnancy affects intestinal mechanical barrier function,^4^, ^5^ intestinal local immune function,^6^ and microecological homeostasis^7^ in the offspring. Emerging research has highlighted the fact that VAD impairs enteric nerve migration, proliferation and differentiation.^8^, ^9^, ^10^ These findings suggested that VAD might cause severe enteric ganglionopathy, including hypoganglionosis, submucosal plexus loss, and abnormal neural differentiation.^10^, ^11^ Our research team has also shown that a VAD diet affects the development of the enteric nervous system (ENS) in the offspring by damaging enteric neurons, which leads to abnormalities in gastrointestinal motility.^12^

Identifying the underlying mechanisms of VAD‐induced intestinal nerve injury might help us to better comprehend the damage to intestinal function caused by VAD. In recent years, it has been revealed that epigenetic control of gene expression patterns can influence a variety of pathological processes, including gut‐associated diseases.^13^, ^14^, ^15^ As one of the most important epigenetic processes, DNA methylation is important in neurodevelopment and is susceptible to environmental factors.^16^, ^17^ It has been shown that a VAD diet in pregnant rats leads to hypermethylation of the promoter regions of heart‐related developmental genes in the offspring and decreases gene expression levels, resulting in embryonic cardiac malformations.^18^ Moreover, VA supplementation in lactating rats caused DNA hyper‐ or hypomethylation of promoters of genes associated with metabolism, thereby affecting the biological properties of white adipose tissue.^19^ Passador's study indicated that the daily intake of calories and/or the micronutrient VA can potentially modulate the global DNA methylation profile of leukocytes in older adults and supports the notion of nutritional influences on epigenetic mechanisms.^20^ These studies have made some progress in understanding the alterations of DNA methylation associated with VA, with implications for embryonic development and differentiation, metabolism, and immune responses.^18^, ^19^, ^20^, ^21^

However, there have been no studies on methylation related to enteric nerve function. Consequently, to understand the specific underlying mechanisms of VAD's harmful effects on the colonic transfer function, in the present study, we first constructed three rat models: VA normal (VAN) and VAD starting from pregnancy, as well as postnatal VA supplementation (VAS), to compare their ENS. Then, transcription sequencing and methylomic analysis of colonic tissue were conducted in the rats from the VAN, VAD, and VAS groups. Lastly, the methylation patterns were combined with transcriptome sequencing results to identify the specific target genes associated with VAD in enteric nervous dysfunction. The study provides a novel perspective to explore the mechanisms by which gestational VAD impairs ENS function, filling the gap of VAD in the epigenetic modification of intestinal transfer function.

## 2 MATERIALS AND METHODS

### 2.1 Animal treatment

Twenty one 3‐week‐old female Sprague Dawley (SD) rats were purchased from Chongqing Medical University, randomly divided into the VAN group (*n* = 7) and the VAD group (*n* = 14), and kept in ventilated cages in the specific pathogen free area of the Experimental Animal Centre of the Children's Hospital of Chongqing Medical University, where temperature and humidity were controlled appropriately, and a daily cycle of light from 7:00 to 19:00 and dark from 19:00 to 7:00 was maintained. One week after the rats were acclimatized to the new environment, the VAN and VAD animal models were constructed as shown in Figure S1. After adaptation to the environment, the rats were randomly divided into maternal VAN and maternal VAD groups, who were fed a VA‐normal diet containing 6500 IU/kg (VAN group) and a VA‐deficient diet containing 400 IU/kg VA (VAD group).^12^ Tail vein serum was collected and retinol concentrations were measured after 4–5 weeks of continuous special chow feeding. Retinol concentrations were greater than 1.05 mmol/L in the maternal VAN rats and less than 0.7 mmol/L in the maternal VAD rats. The maternal rats were then mated 1:1 with males of the same age, and the females in the VAN and VAD groups were fed the VAN and VAD diets, respectively, during gestation, until 3 weeks post‐pup birth. In contrast, the VAS group was established by randomly selecting postnatal pups from the maternal VAD group, replacing the VAD diet with the VAN diet on the first postnatal day (PD), and supplementing the pups with VA by gavage for a period of 7 days, at a supplemental dose of 8.33 IU/g·d. In addition, the VAN diet in the VAS‐fed group was substituted from the birth of the pups, and both the mother and pups were maintained simultaneously until 3 weeks after the birth of the pups. At this time, blood and colonic tissues of 3‐week‐old rats among the offspring of VAN (*n* = 40 from 5 litters of rats), VAD (*n* = 40 from 5 litters of rats), and VAS (*n* = 40 from 5 litters of rats) three groups were collected, snap‐frozen using liquid nitrogen, and stored at −80°C for preservation. In order to eliminate the sexual effect, all offspring rats used for this research were males.

### 2.2 Measurements of serum retinol

Serum retinol levels were measured using high performance liquid chromatography (HPLC) according to a previous study.^12^

### 2.3 Detection of colonic fecal water content

When the offspring of rats in the VAN (*n* = 10 from 2 litters of rats), VAD (*n* = 10 from 2 litters of rats), and VAS (*n* = 10 from 2 litters of rats) three groups were 6 weeks old, 10 rats in each group were randomly selected to test the water content of their feces. Each rat was placed in a cage for 2 h. Fresh feces were collected and weighed immediately, and then heated in an oven at 65°C for 2 h. The dry weight was measured, and the water content was calculated by the difference between the wet and dry weights.

### 2.4 Total GI transport time

Total GI transit time was measured by preparing a carmine solution for gavage, as previously described.^12^

### 2.5 Colon transit time

Distal colonic passage time was measured using a 3 mm diameter glass bead, as described previously.^12^

### 2.6 Transmission electron microscopy

The proximal colon was taken and prefixed with 3% glutaraldehyde, then the tissue was postfixed in 1% osmium tetroxide, dehydrated in an acetone series, infiltrated with Epon 812 for 1 h, and embedded. Semithin sections were acquired, stained with methylene blue, and then ultrathin sections were cut using a diamond knife, followed by staining with uranyl acetate and lead citrate. Sections were examined using JEM‐1400‐FLASH Transmission Electron Microscope (JEOL, Tokyo, Japan). Image J software (NIH, Bethesda, MD, USA) was used to measure the width of the synaptic gap and the length of the postsynaptic density (PSD).

### 2.7 qRT‐PCR and western blotting

Total RNA was extracted from the proximal colon tissue of offspring rats according to the protocol of the RNA Extraction Kit (Promega Biotech, Beijing, China) and subsequently a Prime Script RT Reagent Kit (Takara, Kyoto, Japan) was used to generate cDNA from the mRNA. The qPCR assay was performed using the SYBR‐Green Real‐time PCR Kit (QiaGenes, Germany) and a CFX96 real‐time PCR detection system (Bio‐Rad, Hercules, CA, USA). Primers utilized in this study are shown in Table S2.

Total proteins from the proximal colon tissues were extracted using Radioimmunoprecipitation assay (RIPA) lysis buffer (KGI Bio, Shanghai, China), and quantified using a BCA Protein Assay Kit (KGI Bio, Shanghai, China). The proteins were separated using SDS‐PAGE gel and transferred to 0.45 μm PVDF membranes. After blocking, the membranes were incubated with primary antibodies, washed, and then treated with the corresponding secondary antibodies. The immunoreactive protein bands were detected using the ChemiDoc Imaging System (Bio‐Rad Hercules, CA, USA) and quantitatively analyzed using ImageJ software. The primary antibodies used in this experiment can be seen in Table S3.

### 2.8 Transcriptome sequencing

The RNA sequence analysis method was conducted as described in the previously published study (see Supporting Information S1: Methods).^22^

### 2.9 Representative reduced bisulfate sequencing

The Representative reduced bisulfate sequencing (RRBS) analysis method was described in the Supporting Information S1: Methods.

### 2.10 Bioinformatic analyses

In our research, the results were visualized using Venn diagrams, violin charts, and pie charts, within the OmicStudio platform (https://www.omicstudio.cn/tool). Protein‐protein interaction (PPI) networks were constructed using the STRING database (https://string‐db.org/) and were based on sets of genes with low expression levels and high methylation. Moreover, Cytoscape software (version 3.9.1) was applied to visualize the network. In addition, GEOquery, annotate, gene set enrichment analysis (GSEA, http://software.broadinstitute.org/gsea/ index.jsp) and ReactomePA were used to annotate and enrich the differentially expressed genes (DEGs). All R packages were obtained from Goatools (https://github.com/tanghaibao/GOatools) and the Python scipy package (https://scipy.org/install/). Based on the Gene Ontology (GO) annotation and Kyoto Encyclopedia of Genes and Genomes (KEGG) pathway analysis, DMGs were enriched using DAVID (https://david.ncifcrf.gov/) and Pathview (https://pathview.uncc.edu/). In all the results analysis, *p* values < 0.05 were deemed to indicate significant enrichment.

### 2.11 Statistical analysis

Data are expressed as means ± standard error of the mean (SEM). All data were processed and analyzed using GraphPad Prism software (version 8.0.2, GraphPad Software, Inc., La Jolla, CA, USA) and differences between groups were analyzed using one‐way analysis of variance followed by Tukey's post hoc test. A *p* value < 0.05 was set as a level of statistical significance.

## 3 RESULTS

### 3.1 GI transit dysfunction in offspring rats caused by VAD could be restored by postnatal VAS

**FIGURE 1:**
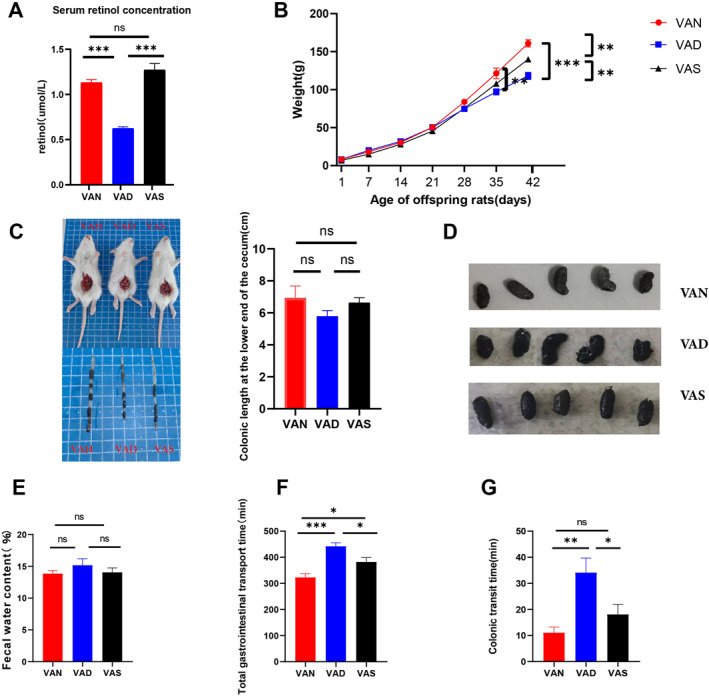
Maternal VA levels during pregnancy affect the weights, colon lengths, morphologies of enteric neurons and GI transmission function in offspring rats. (A) Serum retinal concentrations in 3‐week‐old offspring from the VAN, VAD, and VAS groups (*n* = 10 per group). (B) Body weight curve (*n* = 10). (C) Anatomical observations of the intestines and the length of the colonic segment at the lower end of the cecum of 3‐week‐old offspring from the three groups (*n* = 3). (D) Morphological observation of fresh feces from 6‐week‐old offspring from the VAN, VAD, and VAS groups. (E–G) Comparison of fecal water content, total gastrointestinal transport time, and colonic transit time among the 6‐week‐old offspring from the three groups (*n* = 10). *p < 0.05, **p < 0.01, ***p < 0.001; GI, gastrointestinal; ns, not significant; VA, vitamin A; VAD, vitamin A deficiency; VAN, vitamin A normal; VAS, vitamin A supplementation.

To determine whether we successfully established offspring rat models of VAN, VAD, and VAS, the serum retinol concentrations of these three groups were first measured using HPLC. Serum retinol levels were lower in the VAD group than in the VAN group (*p* < 0.001) and the VAS group (*p* < 0.001), as shown in Figure 1A. Figure 1B shows that the weight loss in the VAD rats compared with that in the VAN rats continued until PD 35 (*p* = 0.0031) and PD42 (*p* < 0.001), respectively. VAS rats had higher body weights than the VAD rats at both PD35 (*p > *0.05) and PD42 (*p* = 0.0021), but still had lower body weights at PD42 compared with the VAN group (*p* = 0.0031). Compared with the VAN and VAS groups, the intestinal tubes of 3‐week‐old VAD rats were mildly distended, and there was a tendency for the length of colonic segment at lower end of cecum to decrease, but there was no significant difference (Figure 1C). The 6‐week‐old VAD rats had mushy, thin stools, while VAN and VAS groups had soft, formed stools (Figure 1D). The percentage of fecal water content was slightly higher in the VAD rats than in the VAN and VAS groups (Figure 1E), but not statistically different among the three groups (*p* > 0.05). The total gastrointestinal transit time was significantly longer in the VAD rats than in the VAN rats (*p* < 0.001) and VAS rats (*p* = 0.0217), while one of the VAS rats was also longer than the VAN rats (*p* = 0.024) (Figure 1F). In addition, the colonic transit time was significantly delayed in the VAD rats compared with those in the VAN (*p* = 0.0014) and VAS groups (*p* = 0.0272) (Figure 1G).

### 3.2 VAD disrupted the transcriptome profile of the colon, affecting genes involved in intestinal nerve function

**FIGURE 2:**
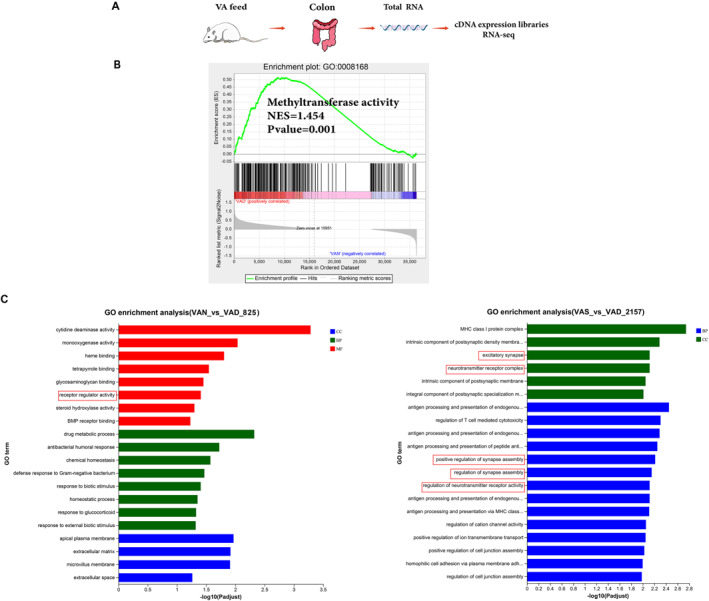
Maternal vitamin A deficiency disrupts transcriptomic profiles of offspring's colon. (A) Experimental design diagram. (B) GSEA on methyltransferase activity between the VAN and VAD groups. (C) GO enrichment analysis of DEGs between the VAN and VAD groups, and between the VAD and VAS groups. *n* = 10 per group; DEGs were identified according to |fold‐change| ≥1.5 and *p* value < 0.05. BP, biological process; CC, cellular component; DEGs, differentially expressed genes; GO, gene ontology; GSEA, gene set enrichment analysis; MF, molecular function.

To assess the possible dysfunction of the colon caused by altered gene transcription as a result of VAD during pregnancy, RNA sequencing of the colon was performed (Figure 2A). GSEA indicated that methyltransferase activity was enriched in the VAD group compared with that in the VAN group (normalized enrichment score = 1.454, *p* value = 0.001, Figure 2B). We found that DEGs between the VAN and VAD groups were enriched in GO molecular functional categories related to enteric nerves, enriched in receptor regulator activity (Figure 2C), and DEGs between the VAS and VAD groups were enriched in the category of biological processes and cellular components related to enteric nerves, and were enriched in pathways such as excitatory synapse, neurotransmitter receptor complex, positive regulation of synapse assembly, regulation of synapse assembly, and regulation of neurotransmitter receptor activity (Figure 2C). The results suggest that the colonic transcriptome profile associated with enteric nerve function is affected by different VA levels.

### 3.3 DNMT1 and DNMT3b expression levels were upregulated in the VAD group

**FIGURE 3:**
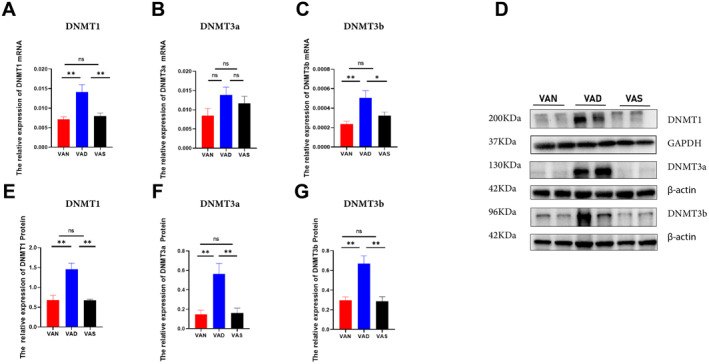
Expression levels changes of DNA methylation‐related enzymes in the colons of 3‐week‐old offspring among the VAN, VAD, and VAS groups. (A–C) The relative mRNA expression levels of Dnmt1, Dnmt3a, and Dnmt3b among the three groups. (nVAN = 9, nVAD = 10, nVAS = 10). (D) Western blotting analysis of DNMT1, DNMT3a, and DNMT3b among the three groups. (E–G) The relative protein levels of DNMT1, DNMT3a, DNMT3b among the groups. (*n* = 4 per group). *p < 0.05, **p < 0.01; DNMT, DNA methyltransferase; ns, not significant.

To investigate whether DNA methylation changes are involved in the development of VAD during pregnancy, we first evaluated the expression levels of DNMTs in the three groups. The mRNA levels of *Dnmt1* and *Dnmt3b*, but not *Dnmt3a*, were increased in the VAD group compared with those in the VAN (*p* = 0.0019 and *p* = 0.004, respectively) and VAS groups (*p* = 0.0048 and *p* = 0.0476, respectively) (Figure 3A–C). The protein expressions levels of DNMT1, DNMT3a, and DNMT3b were higher in the VAD group rats than those in the VAN group (*p* = 0.0021, *p* = 0.0075 and *p* = 0.0033, respectively) and the VAS group (*p* = 0.002, *p* = 0.0094 and *p* = 0.0028, respectively) (Figure 3D–G). Thus, upregulation of DNMTs support the hypothesis that aberrant DNA hypermethylation occurs in the colon of VAD offspring rats.

### 3.4 Hypermethylation in promoter and exon regions by VAD damaged enteric nerve‐related pathways

**FIGURE 4:**
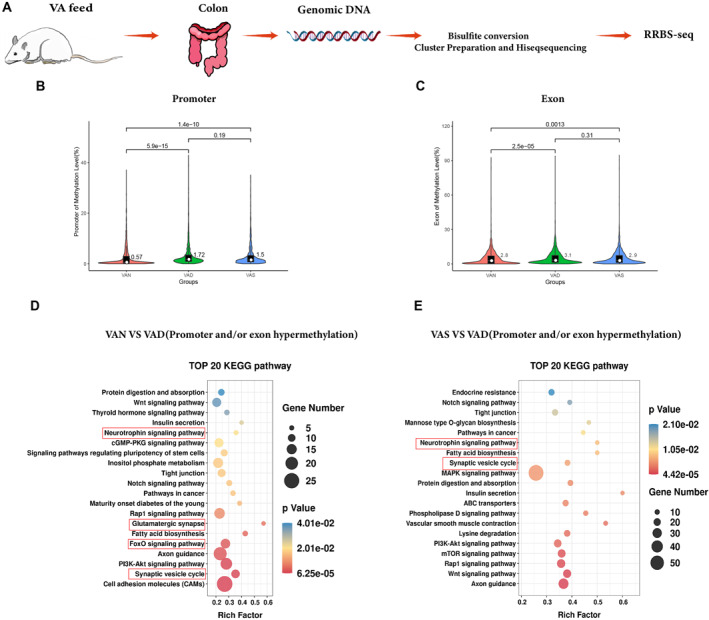
Maternal vitamin A deficiency alters methylation patterns in the offspring's colon. (A) Schematic diagram of representative reduced bisulfate sequencing (RRBS). (B, C) Violin plots showing the total methylation levels for genomic features—including promoters and exons—in the VAN, VAD, and VAS groups. Median methylation levels are indicated by numerical values and white circles. (D, E) Top 20 KEGG pathways of DMR‐associated genes based on the number of DMGs, with the *p* value and rich factor. *n* = 5 per group. DMRs were identified according to a false discovery rate (FDR) < 0.05. DMGs were identified according to hypermethylation in the VAD group compared with both the VAN and VAS groups, |fold‐change| ≥1.2, FDR <0.05. DMGs, differential methylated genes; DMRs, differentially methylated regions.

We next determined the DNA methylome of colons in the three groups via RRBS, which was developed to evaluate the DNA methylation of high‐GC regions at the single base‐pair resolution (Figure 4A). The VAD group had significantly higher methylation levels than the other two groups (Figure 4B,C), consistenting with our observation that DNMT1 and DNMT3b were elevated in VAD. Considering that DNA methylation in gene promoter and exon regions is important for gene transcriptional regulation, we focused on those related genes in the differentially methylated regions (DMRs) that were hypermethylated in their promoters and exons in the VAD group. As shown in Figure 4D–E, the top 20 KEGG pathways enriched in the colon of offspring in the VAN *vs*. VAD groups were: the synaptic vesicle cycle, FoxO signaling pathway, Glutamatergic synapse, and Neurotrophin signaling pathway, which are important pathways related to enteric nerves. In the KEGG pathway enrichment of VAS versus VAD, we also identified the synaptic vesicle cycle and Neurotrophin signaling pathway.

### 3.5 Methylated gene *Sgk1* and FOXO pathways were identified by integrated methylome and transcriptome analysis

**FIGURE 5:**
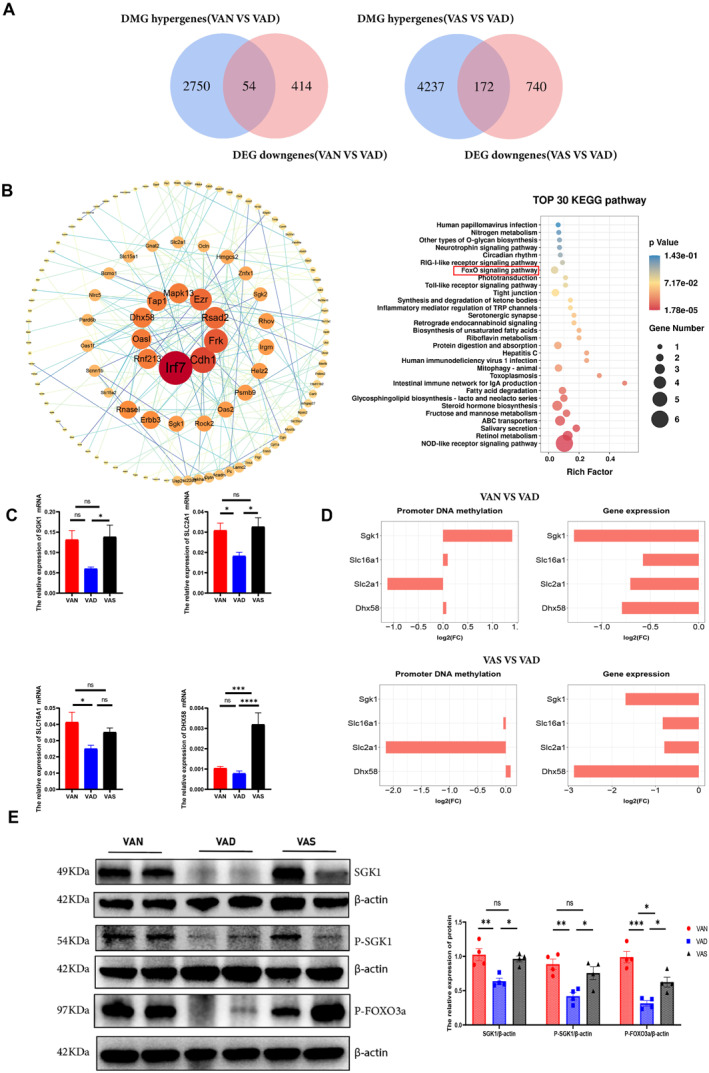
Integration of RRBS and transcriptomic profiles reveals dysregulation of candidate epigenetic genes and networks in maternal vitamin A deficiency‐induced enteric nervous injury. (A) Venn diagrams between the hypermethylated genes related to DMGs and the lower expressed genes related to DEGs in the VAD group compared with VAN or VAS groups. (B) Protein‐protein interaction (PPI) network constructed from overlapped genes in both Venn diagrams. Nodes represent genes and edges represent interactions. The node size represents the node degree. KEGG pathway enrichment of the overlapped genes. Top 30 KEGG pathways are shown in the *Y*‐axis and the *X*‐axis shows the rich factor. (C) Validation of candidate gene expression levels using quantitative real‐time reverse transcription PCR (qRT‐PCR) among the three groups. Data are presented as mean ± SEM (*n* = 10 per group). (D) Log2(fold change [FC]) histogram of promoter methylation levels and expression levels of candidate genes between the VAN and VAD groups and those between the VAS and VAD groups. (E) Western blotting and quantification analysis of serum/glucocorticoid regulated kinase 1 (SGK1) and its downstream protein expression levels among the three groups. (*n* = 4 per group). Significance was tested with one‐way ANOVA. *p < 0.05, **p < 0.01, ***p < 0.001, ****p < 0.0001; ns, not significant.

To identify the potential relationship between DNA methylation changes and gene expression as a result of VAD, an integrated analysis of methylation and transcriptomic profiles was performed. The methylation of promoters and exons is considered to be an important factor influencing gene transcription^23^; therefore, we decided to verify the biological effects of high methylation levels in this region. Using a Venn diagram, we screened a total of 54 genes with low expression and high methylation levels in the VAD versus VAN comparison, and a total of 172 genes in the VAD versus VAS comparison (Figure 5A). The shared genes from the above screen were then used to construct PPI networks and KEGG‐enriched using the STRING database to screen for the top 30 pathways, respectively. In the PPI networks, the proteins with more joint edges have more important biological functions (Figure 5B). We identified four well‐documented markers of immune and nervous system, the molecular types of which mainly included solute carrier family 2 member 1 (SLC2A1), solute carrier family 16 member 1 (SLC16A1), DExH‐box helicase 58 (DHX58) and serum/glucocorticoid‐regulated kinase 1 (SGK1). Figure 5C shows the validation of the expression levels of the four genes in the three groups using qRT‐PCR. *Slc2a1*, *Slc16a1*, and *Sgk1* expression levels were consistent with the results of the transcriptome sequencing data, that is, they all had low levels expressed in the VAD group. Figure 5D shows that only *Sgk1* was identified as hypermethylated and downregulated in the VAD group compared with that in the VAN group. In the VAS group compared with the VAD group, *Sgk1* could not be shown by Log2FC because of the level of DNA methylation in the promoter region of *Sgk1* was zero, however, it still showed hypermethylation and downregulation in the VAD group.

Therefore, the SGK1 and its downstream FOXO signaling pathway, which was also enriched in the Top 20 KEGG pathways in Figures 4, 5 and 4D, were selected for further study. As shown in Figure 5E, the protein level of SGK1 was significantly decreased in the VAD group compared with those in the VAN and VAS groups (*p* = 0.0043 and *p* = 0.0119, respectively). Meanwhile, the level of phosphorylated SGK1 significantly declined in the VAD group compared with those in the other two groups (*p* = 0.0049 and *p* = 0.03, respectively). Notably, the protein level of phosphorylated FOXO3a was significantly lower in the VAD group than the VAN and VAS groups (*p* = 0.0002 and *p* = 0.0259, respectively). We further visualized the methylation levels of CHG, CHH, and CpG of *Sgk1*, and the results indicated that the methylation levels of the representative CpG sites in the promoter and exon regions of DMRs in the VAD group were elevated compared with those in the other two groups (Figure S2).

### 3.6 Sustained VAD during pregnancy undermined colonic neuronal morphology and synaptic structure

**FIGURE 6:**
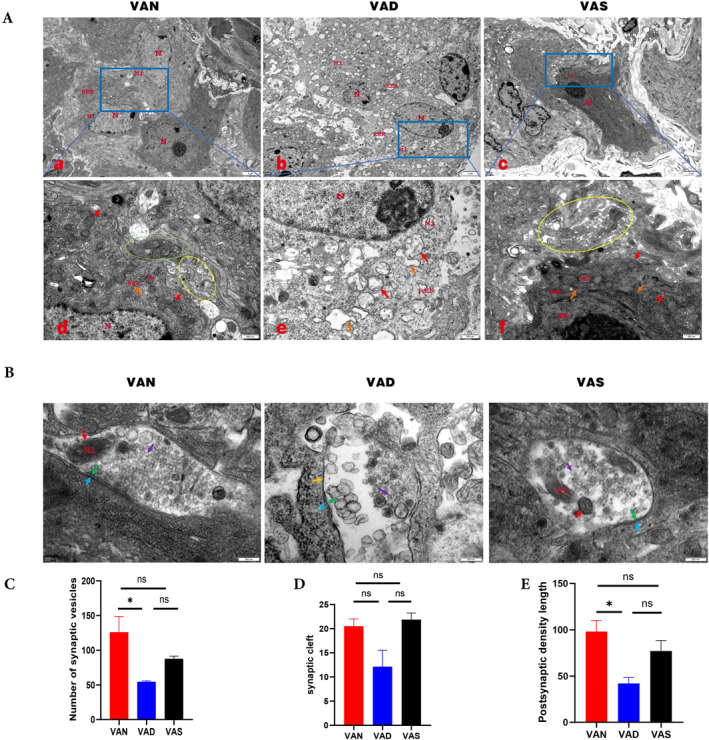
Alterations in the morphology and synaptic structure of three groups of colonic neurons. (A) Observation of neurons within the colonic myenteric plexus by transmission electron microscopy. In the VAN group (a, d), neurons have obvious nucleoli and uniform chromatin distribution; contain a large number of mitochondria, ribosomes, and rough endoplasmic reticulum; have abundant nerve fibers and synaptic structures are also visible around the neuronal cells, where (d) is a diagram of the locally enlarged area of the blue box of (a). In the VAD group (b, e), nucleoli are obvious; mitochondria are swollen; and the rough endoplasmic reticulum is obviously expanded, among which, (e) is a local magnified regional view of the blue box in (b). In the VAS group (c, f), mitochondria and rough endoplasmic reticulum have a more normal morphology and structure; more abundant nerve fibers are also seen around the neurons, as shown in (f), which is a locally enlarged area of the blue box in (c). N, nucleus; Mi, mitochondria (red arrow); RER, rough endoplasmic reticulum (orange arrow); nerve fibers (yellow circle); synaptic structures (green circle). Scale bars: a–c, 2 μm; d–f, 0.5 μm. (B) Ultrastructure of the synapses within the colonic myenteric plexus. Presynaptic membrane (green arrow), synaptic gap (yellow arrow), postsynaptic membrane density (blue arrow), synaptic vesicles (purple arrow), Mi, mitochondria (red arrow); scale bar, 0.2 μm. (C) Number of synaptic vesicles (*n* = 3 per group). (D) Synaptic cleft (*n* = 3 per group). (E) The length of the postsynaptic density (*n* = 3 per group). Values are presented as the mean ± SEM, with one‐way ANOVA for multiple comparisons. *p < 0.05; ns, not significant.

To investigate the effect of VAD on the ENS, we also observed the structure of colonic neurons and synapses in the three groups via TEM. The neuronal structure in the VAD group (Figure 6Ab,e) was poorer than those in the VAN (Figure 6Aa,d) and VAS (Figure 6Ac,f) groups. With quantification the length of the PSD, the synaptic gap, and the number of synaptic vesicles, we found that the number of synaptic vesicles in the VAD group was significantly reduced compared with that in the VAN group (*p* = 0.0398), whereas the number of synaptic vesicles in the VAS group tended to increase compared with that of the VAD group, but without a significant difference (Figure 6C). In contrast, the VAN and VAS groups had relatively normal synaptic gaps, with more intact and clearer dense material on the postsynaptic membrane (Figure 6B). Although there were no significant differences among the three groups, there was a decreasing trend of synaptic cleft width in the VAD group compared with those in the VAN and VAS groups (Figure 6D). In the VAD group, the PSD length was significantly shorter compared with that in the VAN group (*p* = 0.0186), and there was a decreasing trend compared with the VAS group (Figure 6E). The above data suggest that a persistent lack of VA seriously impairs the colonic neurons and integrity of synaptic structure.

## 4 DISCUSSION

The association between VAD and intestinal function has raised extensive public concerns. In recent years, some studies have shown that VAD increases intestinal permeability, which can be ameliorated by VA supplementation.^24^, ^25^, ^26^ Our previous study observed that VA supplementation was effective to ameliorate GI motility in a VAD autistic rat.^27^ Similarly, in the current study, we found that gastrointestinal transit time in VAD littermates was prolonged and could be significantly restored after postnatal VA supplementation.

DNA methylation is one of the important epigenetic mechanisms and correlates negatively with gene transcription.^28^ The effect of nutrients on epigenetics is one of the current research hotspots.^15^, ^17^ We first discovered that the methyltransferase activity was upregulated in the VAD group compared to the VAN group based on GSEA enrichment; further validation confirmed that the mRNA and protein expression levels of DNMT1 and DNMT3b were statistically increased in the VAD group compared to both the VAN and VAS groups. This is consistent with the previous findings of Li et al., who found that low concentrations of plasma retinol and β‐cryptoxanthin might cause macular degeneration by exacerbating the hypermethylation of genes encoding lipid metabolizing enzymes.^29^ In mammalian cells, CpG methylation is accomplished by the classical DNMT1, DNMT3a, and DNMT3b enzymes during epigenetic programming.^30^ DNMT1 is a maintenance methyltransferase that methylates cytosine in hemimethylated CpG dinucleotides,^31^ while the DNMT3 family (DNMT3a and DNMT3b) catalyzes *de novo* methylation of unmethylated CpGs and maintains methylated sites.^32^ Our results showed that VAD altered the DNA methylome of the colon, presenting hypermethylation patterns in promoter and exon regions. To the best of our knowledge, this is the first genome‐wide study to analyze differences in DNA methylation caused by VAD in colonic tissue.

Based on combined RNA‐seq and RRBS data, we validated the expression levels of *Sgk1* gene to investigate the biological effects of hypermethylation in the VAD group, and the data showed that it had significantly synchronized changes in methylation and expression levels (hypomethylated and overexpressed, or hypermethylated and underexpressed). SGK1, as a serine/threonine kinase, participates in the regulation of neuronal excitability, inflammation, proliferation and apoptosis.^33^ SGK1 phosphorylates the Thr‐32 and Ser‐315 sites of FOXO3a, thereby effectively inhibiting FOXO3a‐dependent transcription,^34^ to regulate proliferation and differentiation of colonic enteric neurons.^35^ In our study, the levels of SGK1 mRNA and protein expressions were remarkably downregulated in the VAD group, caused by the higher level of methylation of DMRs in VAD group, as well as attenuation of SGK1 signaling resulted in a diminished protein level of phosphorylated FOXO3a, impaired colonic neuron function of the VAD group. These results hinted that altered epigenetic modifications of *Sgk1* and its downstream impairment of the FOXO3a signaling pathway might be closely related to enteric neuronal development and differentiation.^35^, ^36^

The ENS is an integrated neural network that controls intestinal motility and secretion through multiple types of neurons and complex circuits, mainly including the myenteric plexus and submucosal plexus.^37^ Moreover, enteric nerve injury is not only characterized by dysfunction of peristalsis in the gastrointestinal tract, but also by neuronal deficits and impairment of synaptic function.^12^, ^38^ In the present study, we also observed that the colonic neurons in the offspring of the VAD group displayed the number of synaptic vesicles decreased, and some of the synaptic space atresia and the dense material on the postsynaptic membrane was dissolved. Meanwhile, GO enrichment of DEGs primarily identified neurotransmitter receptor complexes, positive regulation of synapse assembly, and other pathways. The above data point to the fact that the molecular functions of synapses, neurotransmitters, and other molecules associated with enteric nerves were impacted in the VAD group.

The current study could constitute a baseline for future studies focusing on the potential mechanisms of ENS injury‐associated epigenetic regulation caused by VA deficiency. However, it had some limitations. First, considering that colon tissue consists of multiple cell subtypes, future applications of single‐cell technology might provide more accurate results to further determine the underlying mechanisms of VAD‐induced ENS damage. Second, although we identified genes associated with DNA methylation, we acknowledge that these results are largely descriptive and do not establish a causal relationship between DNA methylation and gene transcription levels.

In conclusion, our study indicated that persistent VAD at the start of pregnancy caused elevated methylation levels in colonic tissues, and might trigger downregulation of the SGK1/FOXO signaling pathway, potentially leading to damage colonic neuron and synaptic structure, as well as dysfunctional GI transit in the offspring. However, timely VA supplementation in postnatal offspring successfully upregulated SGK1/FOXO3a signaling axis, which ameliorated the disrupted GI transmission function. This research provides vital information and new investigative ideas for the mechanism by which VA metabolism influences intestinal transit function from an epigenetic perspective.

## AUTHOR CONTRIBUTIONS

Conceptualization: Bei Tong; Data curation: Bei Tong; Formal analysis: Bei Tong, Junyan Yan, Fang Lin, Zhujun Sun and Riqiang Hu; Funding acquisition: Jie Chen; Investigation: Bei Tong; Methodology: Bei Tong, Ruifang Luo, Junyan Yan, Ting Yang and Jie Chen; Resources: Bei Tong and Junyan Yan; Validation: Bei Tong and Junyan Yan; Visualization: Bei Tong; Writing—original draft: Bei Tong; Writing—review and editing: Yuting Wang, Junyan Yan and Jie Chen. All authors have read and agreed to the published version of the manuscript.

## CONFLICT OF INTEREST STATEMENT

The authors declare no conflict of interest.

## ETHICS STATEMENT

The animal study protocol was approved by the Ethics Committee of the Children's Hospital of Chongqing Medical University (CHCMU‐IACUC20200403002, CHCMU‐IACUC20231122004).

## Supporting information

Supporting Information S1

## Data Availability

Research data are not shared.
