# Supplementary material for: Vitamin A deficiency triggers colonic methylation potentially impairing colonic neuron via downregulation SGK1/FOXO pathway
Source: Pediatr Discov. 2024 Jun 14;2(4):e86. doi: 10.1002/pdi3.86 (PMC12118261; doi:10.1002/pdi3.86)

**Figure S1. Schematic diagram of gestational rat model of persistent VA deficiency**

VAN - Vitamin A Normal, serum retinol concentration >1.05 μmol/l.

VAD - Vitamin A deficiency, serum retinol concentration <0.7 μmol/l

VAS - Vitamin A supplements, serum retinol concentration >1.05 μmol/l.

**Figure S1.**


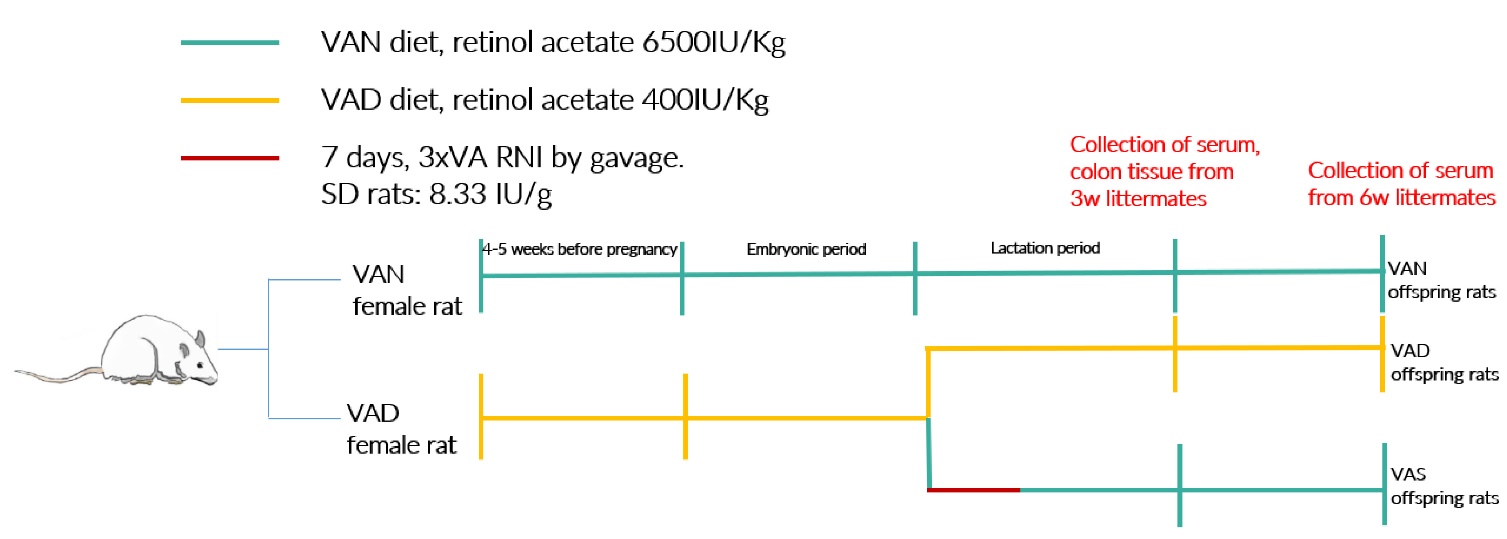

Supplement: Supplementary file 1 — Supporting Information S1 [file PDI3-2-e86-s001.zip › Supporting Information/Figure S1.docx]
