# Supplementary material for: Vitamin A deficiency triggers colonic methylation potentially impairing colonic neuron via downregulation SGK1/FOXO pathway
Source: Pediatr Discov. 2024 Jun 14;2(4):e86. doi: 10.1002/pdi3.86 (PMC12118261; doi:10.1002/pdi3.86)
Supplement: Supplementary file 1 — Supporting Information S1 [file PDI3-2-e86-s001.zip › Supporting Information/Supporting Information S1. methods.docx]

**Transcriptome Sequencing & Database Analysis**

Total proximal colon tissue RNA was extracted from the tissue using the TRIzol® reagent according to the manufacturer’s instructions. Sequencing libraries were constructed using Illumina® Stranded mRNA Prep, Ligation (Illumina, San Diego, CA, USA), based on the Illumina platform, and all mRNAs transcribed from colon tissues were sequenced using an Illumina NovaSeq Reagent Kit. The raw data were quality controlled using fast software (https://github.com/OpenGene/fastp), and then HiSat2 (http://ccb.jhu.edu/software/hisat2/index.shtml) was used to compare the raw data after quality control (clean data [reads]), to the reference genome. Then, the expression levels of genes and transcripts were then quantified separately using RSEM software (http://deweylab.github.io/RSEM/), and finally, DESeq2 software was used for differential expression analysis (*P* value threshold < 0.05) and screening of differentially expressed genes (DEGs). Significant DEGs were screened according to the following criteria: |log2 (fold-change) | > 0.58 & *P*-value < 0.05.

**Representative reduced bisulfate sequencing (RRBS) and Data processing**

Total proximal colon DNA was extracted using a QIAamp Fast DNA Tissue Kit (Qiagen, Dusseldorf, Germany) following the manufacturer's procedure. The DNA samples were fragmented using MspI (NEB, for RRBS) and then subjected to bisulfite conversion. The Accel-NGS Methyl-Seq DNA Library Kit (Swift Biosciences Inc., Ann Arbor, MI, USA) was utilized to attach adapters to the single-stranded DNA fragments. Next, an indexing PCR step was carried out to increase yield and incorporate full length adapters. Bead-based solid-phase reversible immobilization (SPRI) clean-up was used to remove oligonucleotides and small fragments, as well as to change enzymatic buffer composition. Finally, pairwise 2 × 150 bp sequencing was performed on LC Sciences' Illumina Hiseq 4000 platform. Sequence quality was verified using FastQC (http://www.bioinformatics.babraham.ac.uk/projects/fastqc/). Pretreatment of the sequencing data removed more than 5% of reads containing N and low-quality reads (more than 20% of the reads with a mass value of *Q* < 10). A total of 623,902,030 reads were generated, with an average of 41,593469 reads per sample, and an average effective rate of 99.86%. The average proportion of the data quality value greater than 20 was 95.65%; the average proportion of the mass value greater than 30 was 89.47%; and the average content of GC bases was 28.81%. Reads that passed quality control were mapped to reference genome using Bismark.^1^ After alignment, reads were further deduplicated using samtool.^2^ After quality control analyses, a total of 86 Gb of clean reads were generated from 15 libraries (n = 5 per group), and the methylome profile was analyzed based on these results. Differentially methylated regions (DMRs) were analyzed using the R package methylKit,^3^ with a default selection of 1000 bp windows, 500 bp overlap, and a false discovery rate (FDR) and corrected *p*-value < 0.05 as the differential screening threshold for DMRs analysis.

**Reference:**

**1.** Krueger F, Andrews SR. Bismark: a flexible aligner and methylation caller for Bisulfite-Seq applications. *Bioinformatics.* 2011;27(11):1571-1572.

**2.** Li H, Handsaker B, Wysoker A, et al. The Sequence Alignment/Map format and SAMtools. *Bioinformatics.* 2009;25(16):2078-2079.

**3.** Akalin A, Kormaksson M, Li S, et al. methylKit: a comprehensive R package for the analysis of genome-wide DNA methylation profiles. *Genome Biol.* 2012;13(10):R87.
