# Supplementary material for: Vitamin A deficiency triggers colonic methylation potentially impairing colonic neuron via downregulation SGK1/FOXO pathway
Source: Pediatr Discov. 2024 Jun 14;2(4):e86. doi: 10.1002/pdi3.86 (PMC12118261; doi:10.1002/pdi3.86)
Supplement: Supplementary file 1 — Supporting Information S1 [file PDI3-2-e86-s001.zip › Supporting Information/Table S2, Table S3.docx]

**Table S2. Primers used for RT-qPCR.**

**Table S3.** **The primary antibodies used for western blotting.**

| **Table S2. Primers used for RT-qPCR.** | | |
| --- | --- | --- |
| **Gene symbol** | **Forward sequence (5' to 3')** | **Reverse sequence (5' to 3')** |
| *Sgk1* | CCCACGCCAAACCCTCTGAC | GCTTCTTCTGCCTTGTGCCTTG |
| *Dhx58* | CTGCTTCTTCCCTCCTGCTCTG | CCTGAACTCCTGGATCACCTCTTG |
| *Slc2a1* | CGCTTCCTGCTCATCAATCGTAAC | ATCTGCCGACCCTCTTCTTTCATC |
| *Slc16a1* | GCATTGGTGTCATTGGAGGTCTTG | AAGTGGAGCCAGGGTAGAGAGG |
| *Dnmt1* | TGTTCCTCCTTCTGCCATCAATGTG | CATCGTCCTTAGCGTCGTCGTAAC |
| *Dnmt3a* | CGTCACACAGAAGCATATCCAGGAG | CAGGAGGCGGTAGAACTCAAAGAAG |
| *Dnmt3b* | GATGGAGATGGTGAAGCGGATGATG | AGGCTGGAGATACTGTTGCTGTTTC |
| *GAPDH* | ACGGCAAGTTCAACGGCACAG | CGACATACTCAGCACCAGCATCAC |
|  |  |  |
|  |  |  |
|  |  |  |
|  |  |  |

**Table S3.**

| Name | Cat.No., brand | Dilution |
| --- | --- | --- |
| anti-DNA methyltransferase 1 mAb (DNMT1) | 5032;  Cell Signaling Technology, Danvers, MA, USA | 1:1000 |
| anti-DNMT3a mAb | ab188470;  Abcam, Cambridge, MA, USA | 1:1000 |
| anti-DNMT3b mAb | ET1605-9;  HuaBio, Hangzhou, China | 1:1000 |
| anti-serum/glucocorticoid regulated kinase 1 (SGK1) mAb | R381169;  Zenbio, Durham, NC, USA | 1:1000 |
| anri-phospho-SGK1 mAb | 5599S;  Cell Signaling Technology | 1:1000 |
| anti-phospho-Forkhead Box O3a (FOXO3a) pAb | R381118;  Zenbio,  Durham, NC, USA | 1:1000 |
| anti‑glyceraldehyde-3-phosphate dehydrogenase (GAPDH) mAb | HRP60004;  Proteintech, Rosemont, IL, USA | 1:5000 |
| anti-β-actin mAb | AC026;  ABclonal, Wuhan, China | 1:100000 |
