# Supplementary figures and images for: Vitamin A deficiency triggers colonic methylation potentially impairing colonic neuron via downregulation SGK1/FOXO pathway
Source: Pediatr Discov. 2024 Jun 14;2(4):e86. doi: 10.1002/pdi3.86 (PMC12118261; doi:10.1002/pdi3.86)

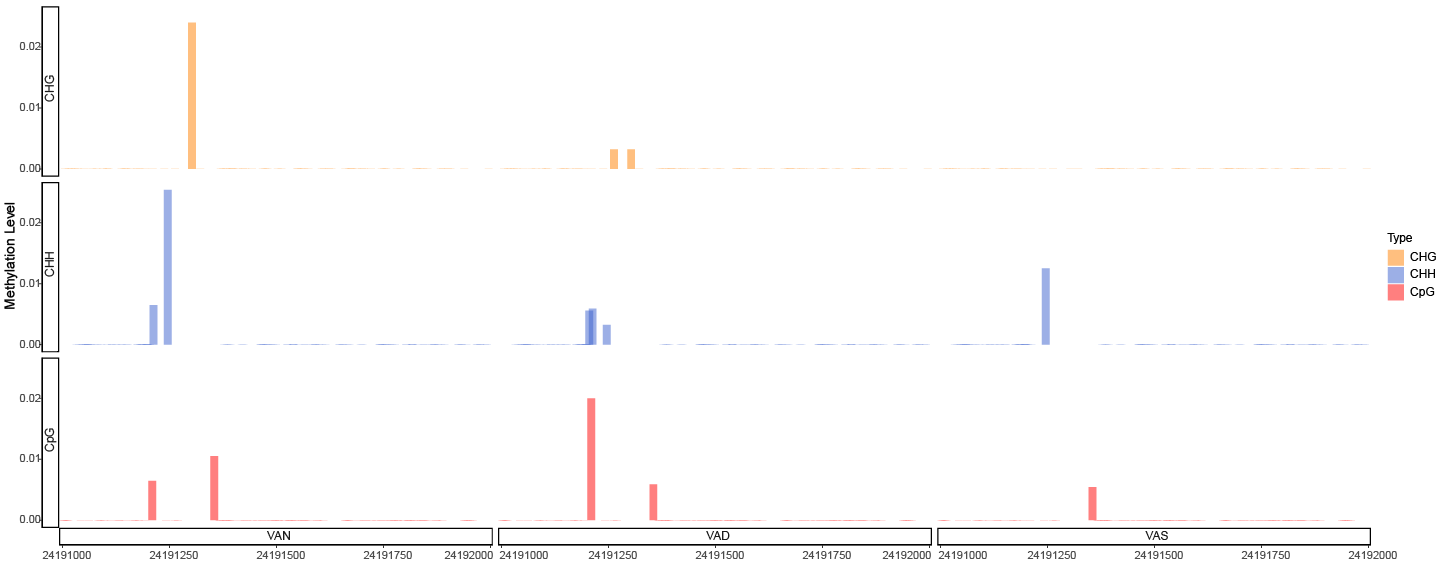

Supplement: Supplementary file 1 — Supporting Information S1 [file PDI3-2-e86-s001.zip › Supporting Information/Figure S2/Figure S2. Sgk1_exon/Figure S2. Sgk1_site_exon methylation.png]

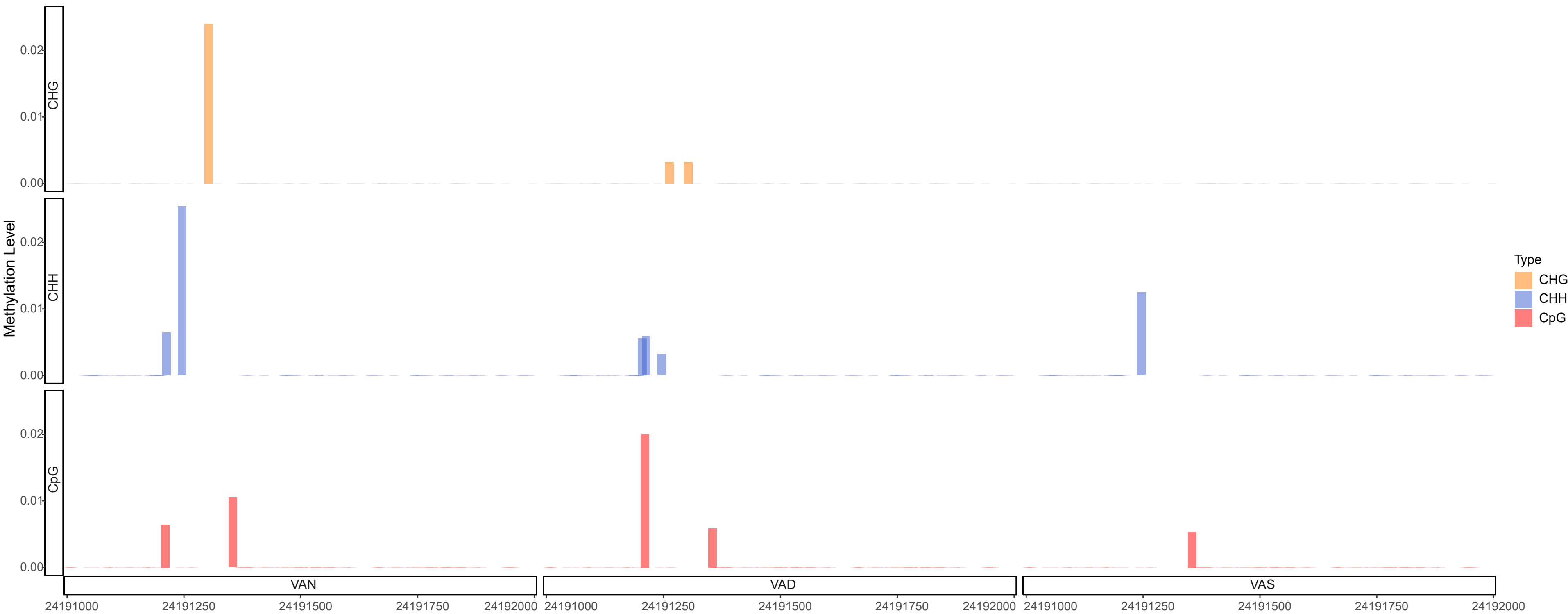

Supplement: Supplementary file 1 — Supporting Information S1 [file PDI3-2-e86-s001.zip › Supporting Information/Figure S2/Figure S2. Sgk1_exon/Figure S2. Sgk1_site_ exon methylation.pdf]

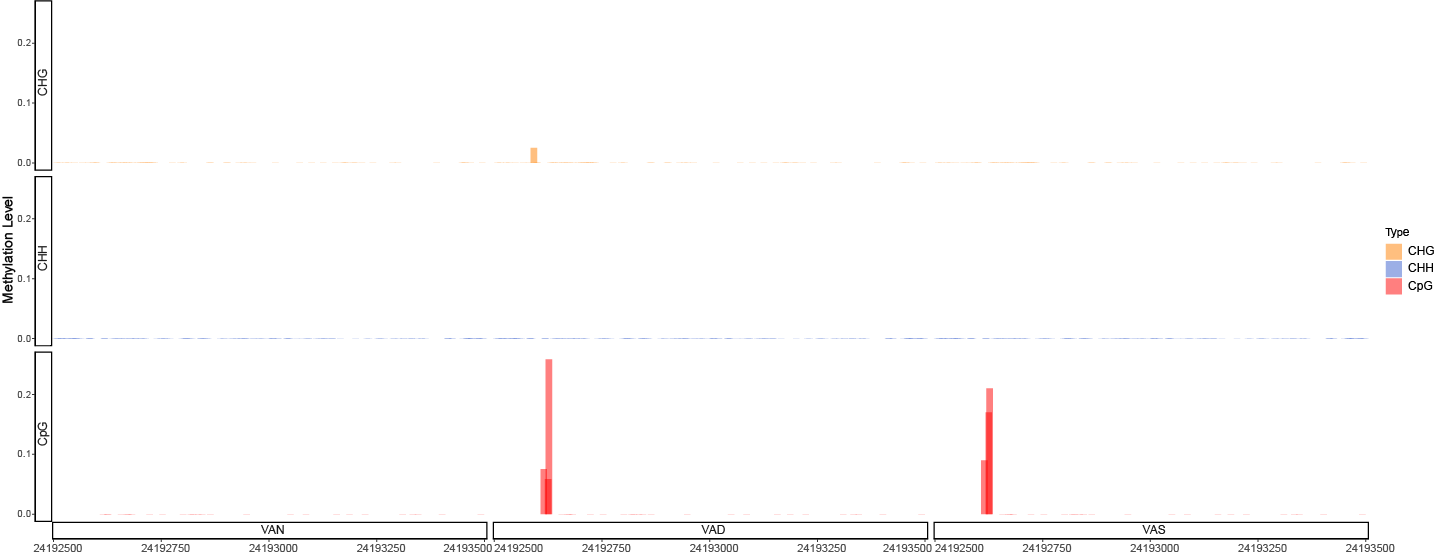

Supplement: Supplementary file 1 — Supporting Information S1 [file PDI3-2-e86-s001.zip › Supporting Information/Figure S2/Figure S2. Sgk1_promoter/Figure S2. Sgk1_site_promoter methylation.png]

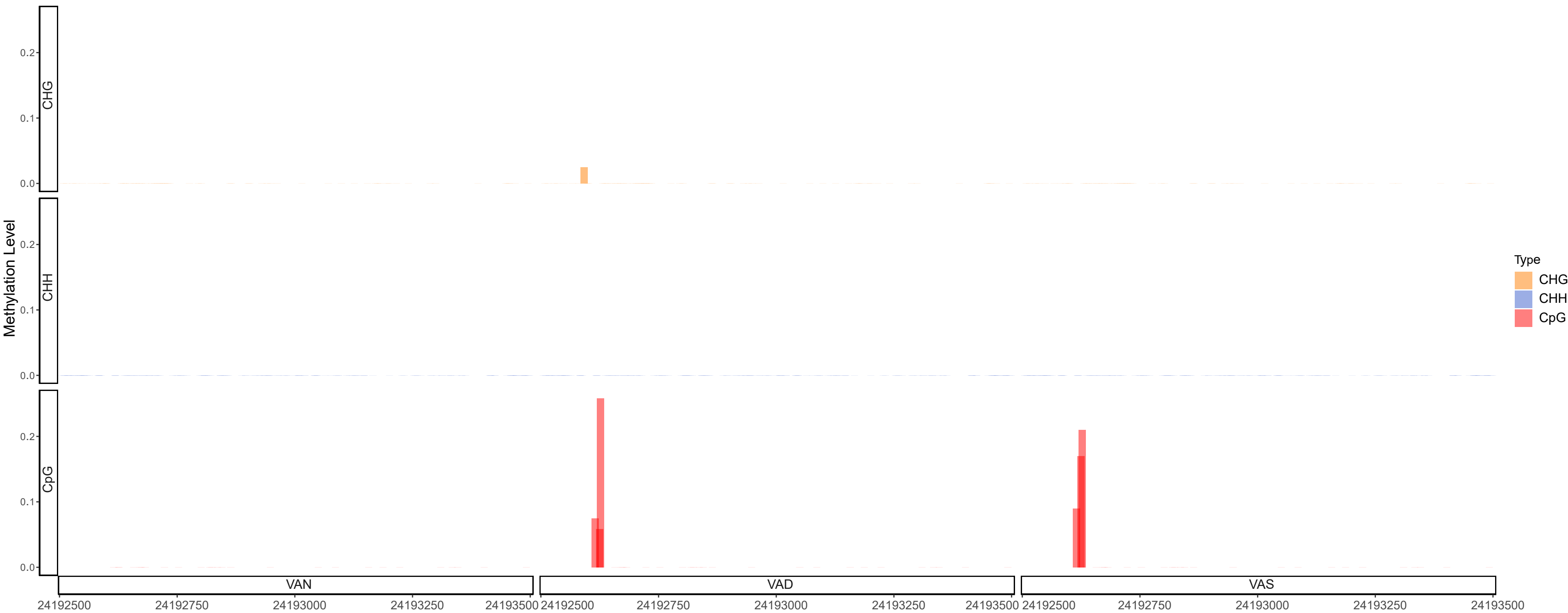

Supplement: Supplementary file 1 — Supporting Information S1 [file PDI3-2-e86-s001.zip › Supporting Information/Figure S2/Figure S2. Sgk1_promoter/Figure S2. Sgk1_site_promoter methylation.pdf]
